# Cadmium passivation induced negative differential resistance in cove edge graphene nanoribbon device

**DOI:** 10.1038/s41598-025-92735-w

**Published:** 2025-03-12

**Authors:** Saurabh Kharwar, Farzan Gity, Paul K. Hurley, Lida Ansari

**Affiliations:** 1 Micro-Nano Systems (MNS) Centre, Tyndall National Institute, University College Cork (UCC), Cork, Ireland; 2 School of Chemistry, University College Cork (UCC), Cork, Ireland

**Keywords:** Electronic devices, Electronic properties and materials

## Abstract

Graphene nanoribbons (GNRs) have emerged as promising candidates for nanoelectronic devices due to their unique electronic and transport properties. In this study, we investigate the impact of passivation on cove-edge graphene nanoribbon (CGNR) using both cadmium (Cd) and hydrogen (H) atoms. Through a comprehensive density functional theory (DFT) analysis coupled with non-equilibrium Green’s function (NEGF) simulations, we explore the electronic transport properties and device behavior of these passivated CGNRs. Our results reveal a distinctive semiconductor-to-metal transition in the electronic properties of the Cd-passivated CGNRs. This transition, induced by the interaction between Cd atoms and the GNR edges, leads to a modulation of the bandstructure and a pronounced shift in the conductance characteristics. Interestingly, the Cd-passivated CGNR devices exhibit negative differential resistance (NDR) with remarkably high peak-to-valley current ratios (PVCRs). NDR is a phenomenon critical for high-speed switching, enables efficient signal modulation, making it valuable for nanoscale transistors, memory elements, and oscillators. The highest PVCR is measured to be 53.7 for Cd-CGNR-H which is x10 and x17 times higher than strained graphene nanoribbon and silicene nanoribbon respectively. These findings suggest the promising potential of passivated CGNRs as novel components for high-performance nanoelectronic devices.

## Introduction

Graphene nanoribbons (GNRs), quasi-one-dimensional (Q1D) structures derived from graphene sheets, have garnered substantial interest in recent years due to their unique electronic, and transport properties^1^. These properties, coupled with their nanoscale dimensions, position GNRs as promising candidates for a wide range of nanoelectronic applications, including transistors, sensors, and quantum devices^2^. The ability to engineer and tailor GNR properties holds the key to unlocking their full potential in next-generation electronic technologies. One significant avenue for tailoring GNR properties is through edge passivation, wherein atoms or molecules are introduced to the ribbon edges to modify their electronic behavior. Edge passivation has been shown to play a crucial role in controlling energy bandgap ($\hbox {E}_g$), electronic states, and transport characteristics in GNRs^3,4^.

Confining graphene in different directions leads to contrasting properties: metallic behavior in the form of zigzag graphene nanoribbons (ZGNRs) when confined in one direction, and semiconducting characteristics as armchair graphene nanoribbons (AGNRs) when confined in the opposite direction^5^. Moreover, these characteristics can be customized according to specific application requirements using different approaches, including doping, edge passivation, application of electric fields along the nanoribbons, and sometimes transverse magnetic fields^6–8^. Cove-edge defected graphene nanoribbons (CGNRs) which are made up of hexagons, were introduced via a bottom-up synthesis approach by Liu et al.^9^. These CGNRs offer a distinctive opportunity for customizing the electrical properties of GNRs to meet various application requirements^10,11^.

Wang et al.^12^ conducted a two-step solution synthesis to create periodic CGNRs using an S-shaped key monomer. Zollo et al.^13^ study delved into a new type of nanoribbon junction in a hybrid cove-edged nanoribbon gap tool tailored for peptide sequencing. They employed transverse tunneling current across the gap while peptides moved through it. The device’s electrode was made from an asymmetric-even cove-edged nanoribbon, feasibly producible using contemporary bottom-up approaches. In a study by Yang and coauthors^14^, a novel type of CGNRs, sulfur-doped CGNRs (S-CGNRs), was synthesized on Au(111) utilizing a specially designed precursor containing thiophene rings. Additionally, Cassiano and colleagues^15^ proposed an edge termination strategy that enables precise tuning of the $\hbox {E}_g$ of CGNRs. This strategy involved a systematic alteration of the periodicity with which armchair-like and zigzag-like edges alternate. Their findings demonstrated that modifications to the edges of CGNRs effectively reduced $\hbox {E}_g$ in a controlled and gradual manner.

Among several methodologies, chemical functionalization emerges as a potent approach to modify the electronic configuration of graphene and its associated nanostructures. Notably, atomic hydrogen is the simplest and most widely used chemical species in this regard. Exposure of graphene to hydrogen leads to a reversible transition from metal-like properties to insulator-like properties^16–18^. Further, the inclusion of Cd atoms has been employed to fine-tune the electronic properties of 2D materials e.g., GNR^19^, aluminum nitride nanoribbons^20^, and ZnO nanosheets^21^. Despite the growing interest in understanding the electronic properties of CGNRs, our current understanding of hydrogen adsorption on CGNRs remains limited. Compared to other GNR configurations, such as AGNRs and ZGNRs nanoribbons, CGNRs offer distinctive electronic properties that can be finely tuned through edge manipulation. This versatility is largely due to CGNRs’ alternating edge geometry, which allows for a higher degree of functionalization and electronic modulation. However, passivating GNRs to achieve desirable electronic properties presents significant challenges. Common issues include controlling edge reactivity, achieving uniform passivation, and preventing structural degradation. Passivation efforts, such as hydrogenation, are effective but often limited in tuning specific properties such as $\hbox {E}_g$ or conductivity in a predictable manner.

In this study, we addresses these challenges by edge passivation on CGNR-a specific GNR configuration known for its distinct structural and electronic properties. In particular, we investigate the impact of hydrogen (H-) and cadmium (Cd-) passivation on CGNRs using a combination of density functional theory (DFT) calculations and non-equilibrium Green’s function (NEGF) simulations. The choice of Cd-passivation stems from its potential to induce significant changes in the electronic structure of CGNRs due to its distinct electronic properties and coordination behavior. H-passivation, on the other hand, is well-known for its ability to modulate $\hbox {E}_g$ and transport properties in GNRs^22^. Our study aims to shed light on how the introduction of Cd and H atoms to the edges of CGNRs influences their electronic properties and, subsequently, their device behavior. We uncover a semiconductor-to-metal transition in the electronic structure of Cd-passivated CGNRs, driven by the interaction between Cd atoms and the GNR edges. This transition gives rise to novel transport characteristics that have potential implications for nanoelectronic device design. Furthermore, we explore the electronic behavior of CGNRs with dual passivation-where both Cd and H atoms are present on opposing edges. Our findings reveal intriguing device properties, including negative differential resistance (NDR) with remarkably high peak-to-valley ratios (PVCRs) and the emergence of multiple PVCR regions. These characteristics offer promising potential for high-speed switching applications, efficient signal modulation, and enhanced functionality in nanoscale transistors, memory elements, and oscillators^23,24^.

The manuscript’s structure is as follows: In “Methods” Section, the computational approach is discussed, “Discussion” Section provides elaboration on the obtained simulation results, and Section “Conclusion” offers the concluding comments.

**Fig. 1:**
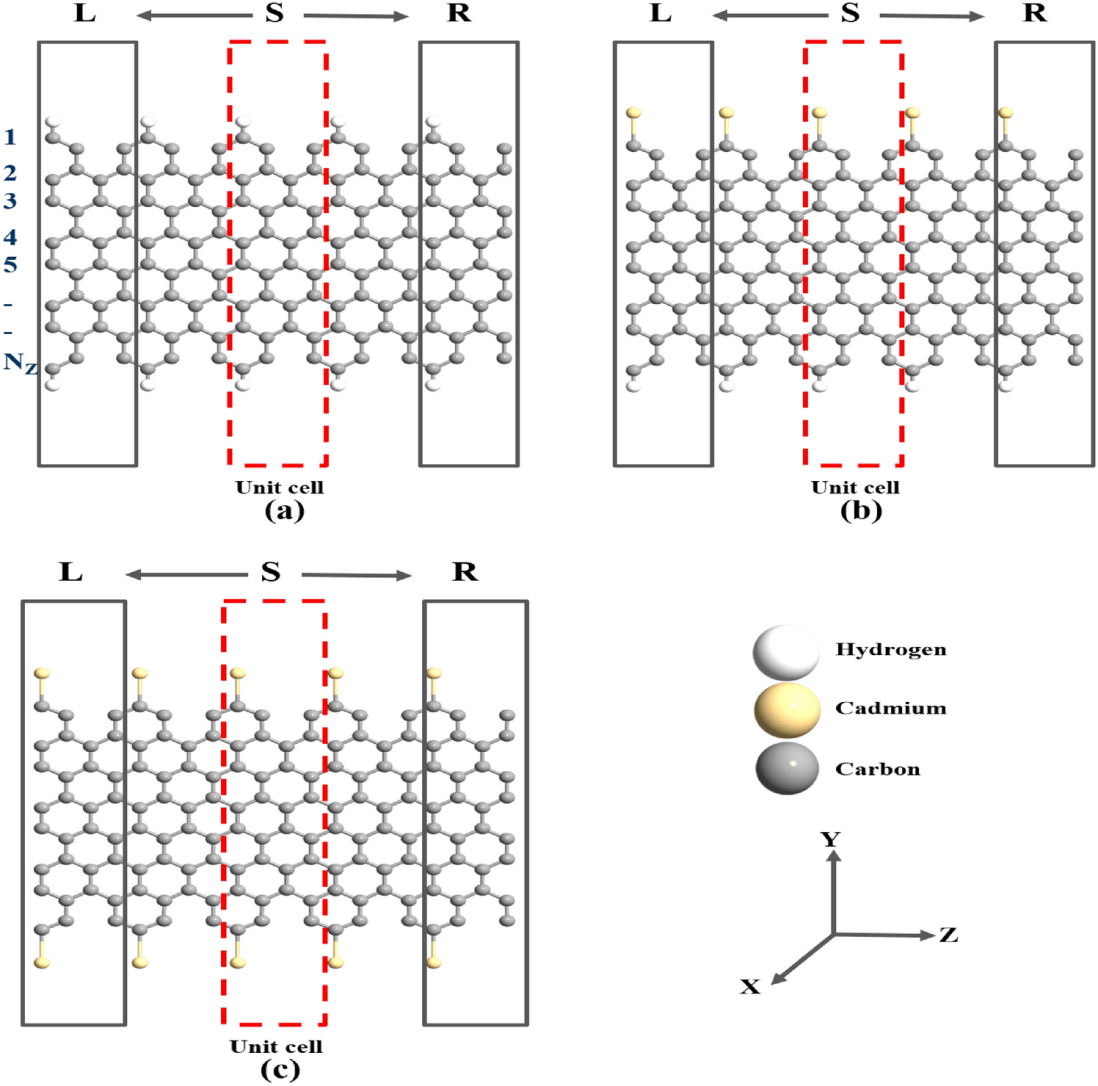
Schematic illustration of CGNRs with $\hbox {N}_Z$=8 : (**a**) H-CGNR-H, (**b**) Cd-CGNR-H, and (**c**) Cd-CGNR-Cd, respectively. The considered device consists of electrodes at the left and right sides shown by black rectangular boxes and scattering region (S) at center. The red rectangular box shows the unit cell.

## Methods

In this study, we employed a comprehensive computational approach to investigate the electronic transport properties of various CGNR configurations, including hydrogen (H) passivation on both edges (H-CGNR-H), cadmium and hydrogen (Cd-H) dual passivation (Cd-CGNR-H), and cadmium (Cd) passivation on both edges (Cd-CGNR-Cd), as shown in Fig. 1. The electronic transport calculations were performed using the DFT and NEGF formalism, as implemented in QuantumWise ATK software^25^. The Local Density Approximation (LDA) exchange-correlation functional was selected for the electronic structure calculations. This choice has been demonstrated to yield accurate results for graphene-based systems^26^. To further clarify, we performed lattice optimization using both LDA and Generalized Gradient Approximation (GGA) for graphene unit cell. The results indicated that the lattice constant obtained using LDA was 2.456 Å, while that from GGA was 2.47 Å. Taking into account the reported experimental values of the lattice constant for graphene^27–31^, this comparison demonstrates that LDA provides a more accurate lattice constant, supporting its selection for the current study. Furthermore, the LDA functional has been shown to provide a more accurate description of edge-passivated graphene nanoribbons^32^.

For all calculations, a $1\times 1\times 100$ k-point grid was used to sample the Brillouin zone, ensuring accurate representation of the electronic structure^33^. The mesh cut-off is set at 75 Ha. The basis sets for the atoms are all double-$\zeta$ polarized^34,35^. A force convergence criterion of 0.05 eV/Å was employed to ensure accurate geometry optimization. To mitigate potential interactions between periodic images, a vacuum padding of 15 Å^36^ and 30 Å are introduced along the edge passivated site (Y-direction) and direction perpendicular to the GNR plane (X-direction) respectively. Structural stability was assessed using the binding energy ($\hbox {E}_b$) calculation, defined as follows:

1 $$\begin{aligned} E_{b}=\frac{E_T-E{_{bare}}-N_{Cd}E_{Cd}-N_{H}E_{H}}{N_{Cd}+N_H} \end{aligned}$$

Here, $E_T$, $E_{bare}$, $E_{Cd}$, and $E_{H}$ are the total energy of passivated structure, bare structure, isolated Cd atom and individual H atom, respectively. $N_{Cd}$, and $N_{H}$ denotes the total number of Cd, and H atoms in the structure. The quantum conductance, described using the Landauer formula, relates the conductance (*G*) of a nanoscale system to the electron transmission probability (*T*(*E*)) at a given energy. The conductance is given by:

2 $$\begin{aligned} G = G_0 \cdot T(E_F), \end{aligned}$$

where $G_0 = \frac{2e^2}{h}$ is the quantum of conductance, *e* is the electron charge, *h* is Planck’s constant, and $T(E_F)$ is the transmission at the Fermi energy ($E_F$). The transport mechanism are explored by Landauer-B$\ddot{u}$ttiker equations^25,34–37^:

3 $$\begin{aligned} G_C(E)= & [{EI-H-\Sigma _{L}^{r}(E)-\Sigma _{R}^{r}(E)}]^{-1} \end{aligned}$$

4 $$\begin{aligned} T(E)= & T_r[\tau _R(E)G_C(E)\tau _L(E)G_C^+(E)] \end{aligned}$$

5 $$\begin{aligned} I(V_b)= & \frac{2e^2}{h}\int _{\varepsilon _L}^{\varepsilon _R}T(E,V_b)[F(E-\varepsilon _L)-F(E-\varepsilon _R)]dE \end{aligned}$$

6 $$\begin{aligned} F_{L,R}(E)= & \frac{1}{\exp \left( \frac{E - \varepsilon _{L,R}}{k_B T}\right) + 1} \end{aligned}$$

where the variables $\hbox {G}_C$(E) and $\hbox {G}_C^+$(E) represent the retarded Green’s function, advanced Green’s function of the channel region. T(E) and I($\hbox {V}_b$) represent the transmission coefficient and current at the applied bias voltage ($\hbox {V}_b$). The Hamiltonian and identity matrices for the retarded Green’s function are denoted by *H* and *I*, respectively. $k_B$ is Boltzmann’s constant, and $\varepsilon _L$ and $\varepsilon _R$ are the electrochemical potentials of the left and right leads, shifted by the applied bias voltage ($eV = \varepsilon _L - \varepsilon _R$). The self-energies and coupling coefficients of the left/right electrodes are denoted by $\Sigma _{L/R}^{r}$ and $\tau _{(L/R)}$ respectively^25,37^.

## Discussion

### Structural properties

The investigation of the structural properties of the considered CGNRs following geometric optimization has provided valuable insights into their stability, bond characteristics, and electronic behavior. The analysis encompasses three distinct configurations: H-CGNR-H, Cd-CGNR-H, and Cd-CGNR-Cd. Geometric optimization of the CGNRs revealed the adoption of a planar structural configuration. The optimization process resulted in minimal deviations from planarity, indicating the inherent stability of the systems.

**Table 1:** Geometrical optimized bond lengths of C–C, Cd-C, and Cd-H in the studied CGNR nanostructures with $\hbox {N}_{{Z}}$=8.

Structure	Bond length (Å)				
	C–C			Cd-C	C-H
	Center	Top	Bottom		
H-CGNR-H	1.41	1.43	1.43	–	1.10
Cd-CGNR-H	1.41	1.36	1.43	2.28	1.10
Cd-CGNR-Cd	1.41	1.36	1.36	2.28	–

**Table 2:** Binding energy ($\hbox {E}_b$), Fermi energy ($\hbox {E}_F$), and bandgap energy ($\hbox {E}_g$) of considered CGNRs.

Width ($\hbox {N}_Z$)	Structure	$\hbox {E}_b$ (eV)	$\hbox {E}_F$ (eV)	$\hbox {E}_g$ (eV)
8	H-CGNR-H	− 5.60	− 5.00	0.14
	Cd-CGNR-H	− 3.38	− 4.13	M
	Cd-CGNR-Cd	− 1.15	− 3.89	M
6	H-CGNR-H	− 5.63	− 4.68	0.32
	Cd-CGNR-H	− 3.41	− 4.15	M
	Cd-CGNR-Cd	− 1.17	− 3.85	M
4	H-CGNR-H	− 5.65	− 4.97	0.71
	Cd-CGNR-H	− 3.43	− 4.10	M
	Cd-CGNR-Cd	− 1.19	− 3.71	M

*Here, M represents metal

The optimized bond length of the Cd and carbon (Cd-C) and H and carbon (H-C) are found to be 2.28 Å and 1.10 Å respectively. The obtained Cd-C and H-C bond lengths are consistent with existing literature, where bond lengths of Cd-C and C-H have been reported as 2.22 Å/2.328 Å^38,39^ and 1.10 Å^37,40^ respectively. This alignment with prior research strengthens the reliability of our findings and provides a useful comparison for understanding the bond length characteristics in the context of our work. Notably, the bond lengths between Cd-C in the Cd-CGNR-Cd configuration were observed to be elongated relative to the initial bond lengths in the bare GNR, a phenomenon consistent with the introduction of the Cd atoms^20^.

Table 1 reports the obtained bond lengths of Cd-C, and H-C after performing post-structural optimization on the CGNRs configurations for width ($\hbox {N}_Z$) = 8. The C-C bond lengths at the center and edge sites were restored after optimization, leading to a slight amount of local reconstruction in the nanoribbons. Structural modifications resulting from differences in bond lengths significantly affect the stability of nanoribbons, as reported by Yogi et al.^41^. Their study found that the bond lengths reduces from 1.43 Å to 1.36 Å for $\hbox {N}_Z$ = 8 due to modifications, indicating that cove-C-edge sites of the nanoribbons underwent more significant reconstructions. This bond length elongation due to edge passivation is consistent with the findings of a previous study^42^, suggesting stronger electron-electron interactions between C and Cd atoms. Importantly, all the configurations studied retained their planar geometries, even after H/Cd passivation.

**Fig. 2:**
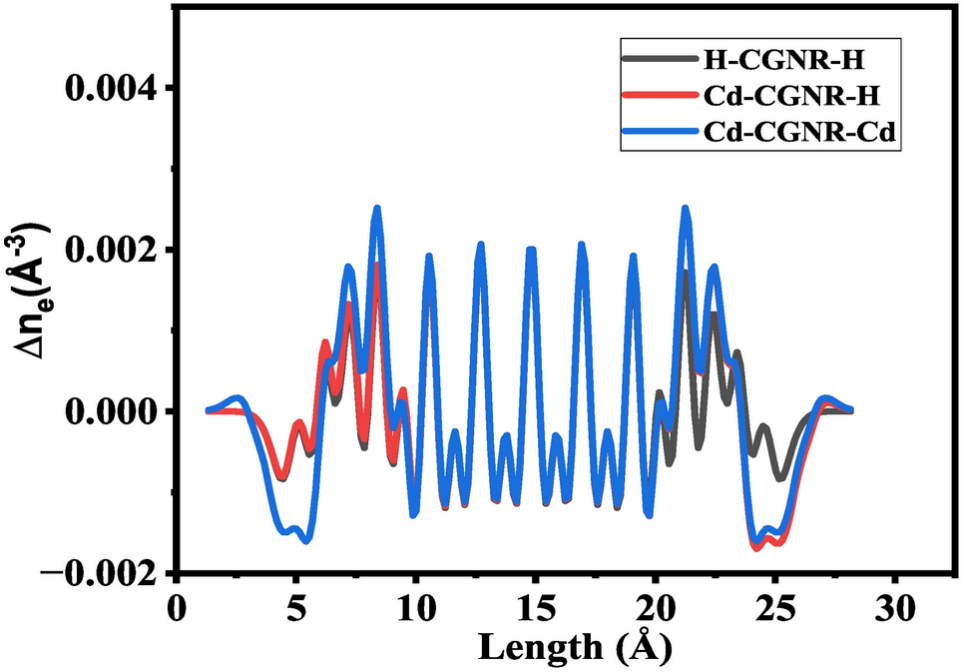
The calculated charge difference density for the H-CGNR-H, Cd-cgnr-H, and Cd-CGNR-Cd for $\hbox {N}_Z$=8.

To assess the stability of the different CGNR configurations, the $\hbox {E}_b$ are calculated as described in the “Methods” section and listed in Table 2. The calculated $\hbox {E}_b$ provide valuable information about the stability of the passivated systems. Remarkably, the Cd-CGNR-H configuration exhibited the more negative binding energy among the studied configurations, indicating enhanced stability compared to the Cd-CGNR-Cd. This observation aligns with the elongation of Cd-C bonds and underscores the role of Cd passivation in reinforcing the CGNR structure. The $\hbox {E}_b$ obtained were further found to correlate with the widths of the GNRs. As anticipated, Cd-CGNR-H, with the narrowest width, displayed the most favorable $\hbox {E}_b$, followed by Cd-CGNR-Cd configurations. This consistency highlights the interplay between width, passivation, and $\hbox {E}_b$, reaffirming the stabilizing influence of Cd atoms. The $\hbox {E}_b$ becomes more negative from $\hbox {N}_Z$ = 8 to 4, suggesting a stronger interaction between the passivating atoms and the graphene edges as the system becomes more confined. The Fermi energy ($\hbox {E}_F$) was explored to gain insights into the electronic characteristics of the CGNRs. Intriguingly, the addition of Cd atoms to the CGNR edges induced a shift in the $\hbox {E}_F$, leading to a characteristic change in the electronic behavior as listed in Table 2. The $\hbox {E}_F$ value measured for H-CGNR-H, Cd-CGNR-H, and Cd-CGNR-Cd is approximately -5.00 eV, -4.13 eV, and -3.89 eV, respectively. The obtained $\hbox {E}_F$ of Cd-CGNR-Cd is approximately equal to the previous study with Cd-ZGNR-Cd^39^. Specifically, an increase in the number of Cd atoms resulted in an upward shift of $\hbox {E}_F$, indicative of a transition toward n-type conductivity. To investigate the effect of passivation on the $\hbox {E}_F$ shift, we calculated the charge difference density (CDD) for H-CGNR-H, Cd-CGNR-H, and Cd-CGNR-Cd, as presented in Fig. 2. The results reveal that the $\hbox {E}_F$ shifts upward with increase in number of Cd atoms, which can be attributed to variations in the CDD, as shown in Fig. 2. Our analysis indicates that Cd-passivated edges exhibit a more significant charge redistribution and a negative CDD compared to H-passivated edges, suggesting electron donation from Cd atoms to the graphene.

Additionally, the Cd atom, being less electronegative than hydrogen, contributes to an upward shift in the Fermi level. This behavior is consistent with previous study with H and F passivation^43^, which have shown that less electronegative atoms, such as Cd, tend to donate electrons to the system, thereby enhancing conductivity. This mechanism may have important implications for potential device applications.

**Fig. 3:**
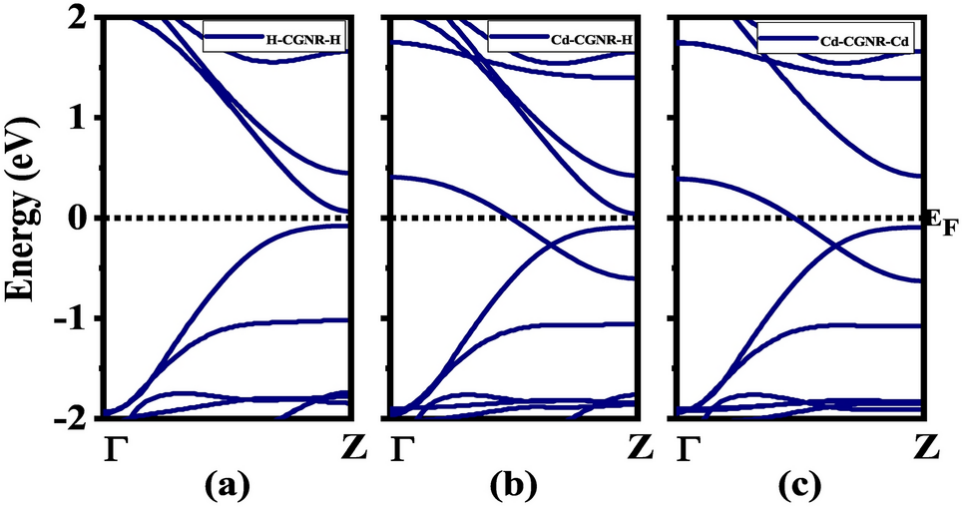
Band structures of considered CGNRs for $\hbox {N}_Z$=8: (**a**) H-CGNR-H, (**b**) Cd-CGNR-H, and (**c**) Cd-CGNR-Cd, respectively. The black-dotted line indicates the Fermi level at 0 eV.

**Fig. 4:**
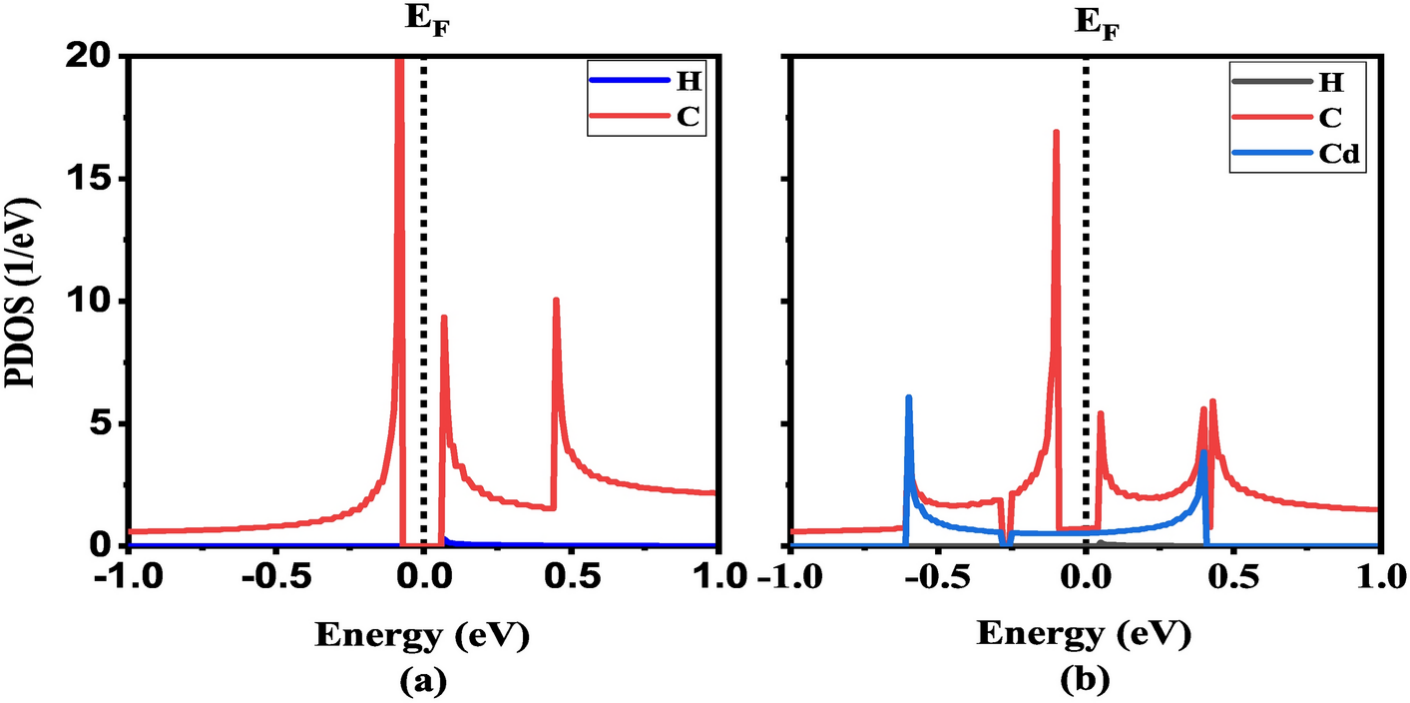
Projected density of states of (**a**) H-CGNR-H and (**b**) Cd-CGNR-H. The black-dotted line indicates the Fermi level at 0 eV.

### Electronic properties

The exploration of electronic properties in the considered CGNRs is paramount to understanding their behavior and potential for electronic applications. In this section, we present an analysis of the electronic band structure and projected density of states (PDOS) for the three distinct configurations: H-CGNR-H, Cd-CGNR-H, and Cd-CGNR-Cd as shown in Figs. 3 and 4 respectively.

The electronic band structure provides crucial insights into the energy levels and electronic states of the CGNRs. As depicted in Fig. 3, the electronic band structures of the three configurations exhibit distinctive features resulting from edge passivation and the presence of Cd atoms. The quantum confinement effect is a key factor influencing the electronic properties of GNRs as the width decreases^44^. To better illustrate this, we have included calculations of the $\hbox {E}_g$ for different GNR widths ($\hbox {N}_Z$ = 8 , 6 , and 4) in the Table 2. In the analysis of Fig. 3a, it is observed that H-CGNR-H demonstrates semiconductor behavior with an $\hbox {E}_g$ of 0.14 eV for $\hbox {N}_Z$ = 8. The H-CGNR-H exhibits finite $\hbox {E}_g$ due to saturation of dangling bond at the edges. The calculated $\hbox {E}_g$ of H-CGNR-H for $\hbox {N}_Z$ = 8 to 6 are observed to increase from 0.14 eV to 0.71 eV which is due to quantum confinement effect. These results underscore the role of quantum confinement in modulating the electronic structure of GNRs and emphasize the importance of width in determining the material’s electronic properties.

Notably, the introduction of Cd passivation (in either Cd-CGNR-H or Cd-CGNR-Cd configuration) induces a transition in the structure’s behavior from semiconducting (when passivated solely with H) to metallic. This transition arises from the interaction between Cd atoms and the GNR edges, leading to the creation of additional electronic states within the $\hbox {E}_g$. Furthermore, the PDOS of H-CGNR-H and Cd-CGNR-H are explored to observe the interaction between Cd and C atoms as shown in Fig. 4. The perusal of Fig. 4a exhibits $\hbox {E}_g$ which is consistent with their bandstructure study. Moreover, the presence of Cd atom introduces additional energy levels near the Fermi energy as observed in Fig. 4(b), influencing the electronic states within the $\hbox {E}_g$ and contributing to the observed changes in electronic behavior.

### Transport properties

The NEGF formalism has been utilized for the exploration of electronic transport characteristics in Cd-passivated CGNR devices. Fig. 1 showcases the two-probe device configurations for the CGNRs under consideration. The considered device consists of electrodes at the left and right sides shown by black rectangular boxes and scattering region (S) at center. The length of region “S” measures approximately 14.78 Å, while each electrode spans 4.92 Å.

**Fig. 5:**
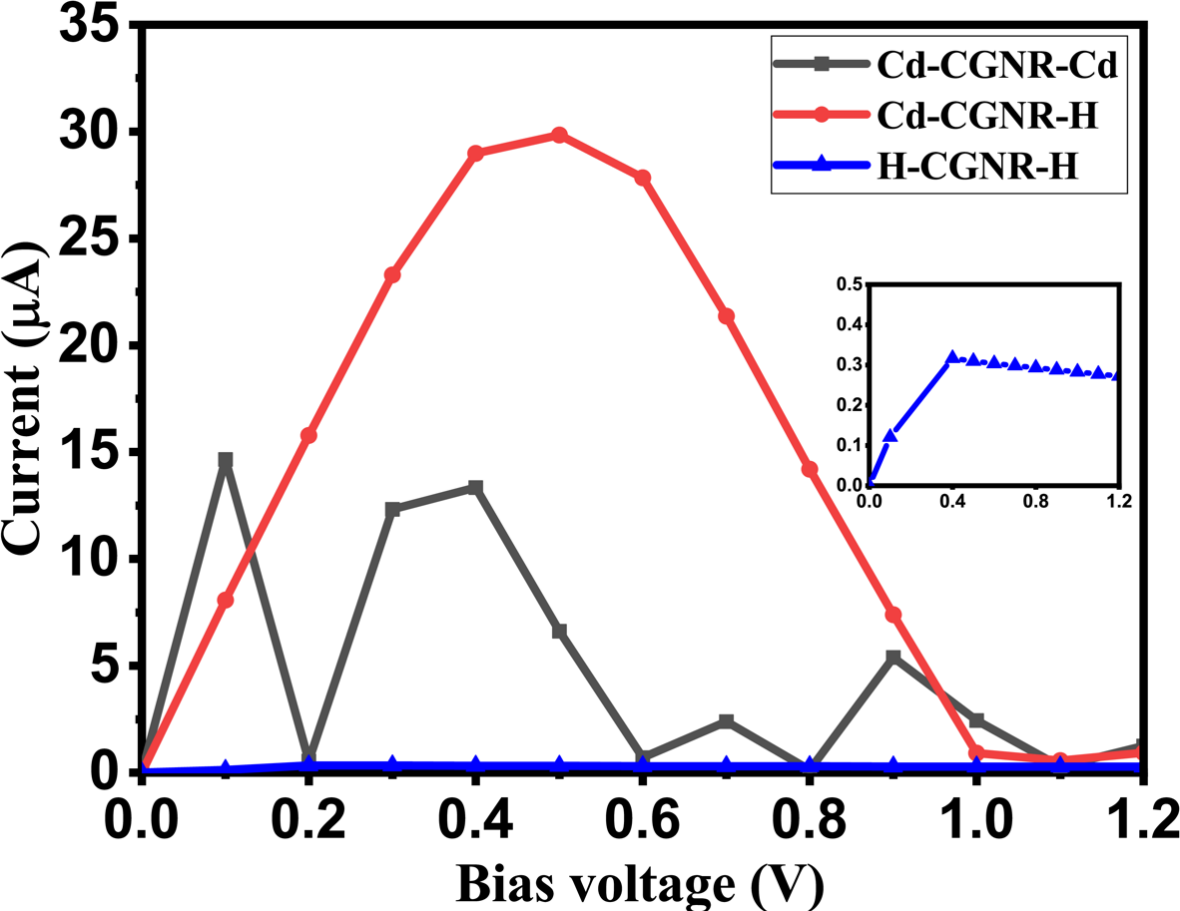
I–V curves for nanodevices based on H-CGNR-H, Cd-CGNR-H, and Cd-CGNR-Cd, for $\hbox {N}_Z$=8.The inset of Fig. shows the I-V of H-CGNR-H.

The current-voltage (I–V) characteristics calculated for the specified CGNRs are depicted in Fig. 5. Analysis of the I–V characteristics has been conducted across a range from 0 to 1.2 V. It’s observed that electron transmission is impeded across the nano-junction in both edge H-passivated CGNRs (H-CGNR-H). This results in a notably low current when an external finite voltage is applied due to its $E_g$. The Cd-CGNR-Cd and Cd-CGNR-H devices, utilizing cove-edge Cd-CGNRs, display the NDR effect as seen in Fig. 5. Specifically, the Cd-CGNR-H device exhibits NDR characteristics across a wide $\hbox {V}_b$ range, while the Cd-CGNR-Cd device shows NDR with multiple peaks in a lower $\hbox {V}_b$ range.

The current peaks at $\hbox {V}_b$ of 0.5 V for Cd-CGNR-H and 0.1 V for Cd-CGNR-Cd devices. Subsequently, it declines until $\hbox {V}_b$ reaches 1.1 V for Cd-CGNR-H and 0.2 V for Cd-CGNR-Cd, showcasing the NDR traits. The corresponding PVCR values are provided in Table 3, calculated using the formula specified in Eq. (5)^42^:

7 $$\begin{aligned} PVCR = \frac{I_{Peak}}{I_{Valley}} \end{aligned}$$

Here, $\hbox {I}_{{Peak}}$ denotes the highest achieved current at the peak voltage ($\hbox {V}_{{Peak}}$), while $\hbox {I}_{{Valley}}$ indicates the minimum current observed after the maximum current at the valley voltage ($\hbox {V}_{{Valley}}$). The calculated PVCR values stand at 53.7 for the Cd-CGNR-H device and 27 for the Cd-CGNR-Cd device.

**Table 3:** Peak-to-valley current ratio (PVCR), along with the corresponding peak voltage, peak current, valley voltage, and valley current, for the considered Cd-CGNR devices.

	Cd-CGNR-H	Cd-CGNR-Cd
$\hbox {V}_{{Peak}}$ (V)	0.5	0.1
$\hbox {I}_{{Peak}}$ ($\mu$A)	30	14.7
$\hbox {V}_{{Valley}}$ (V)	1.1	0.2
$\hbox {I}_{{Valley}}$ ($\mu$A)	0.56	0.54
PVCR	53.7	27.22

**Fig. 6:**
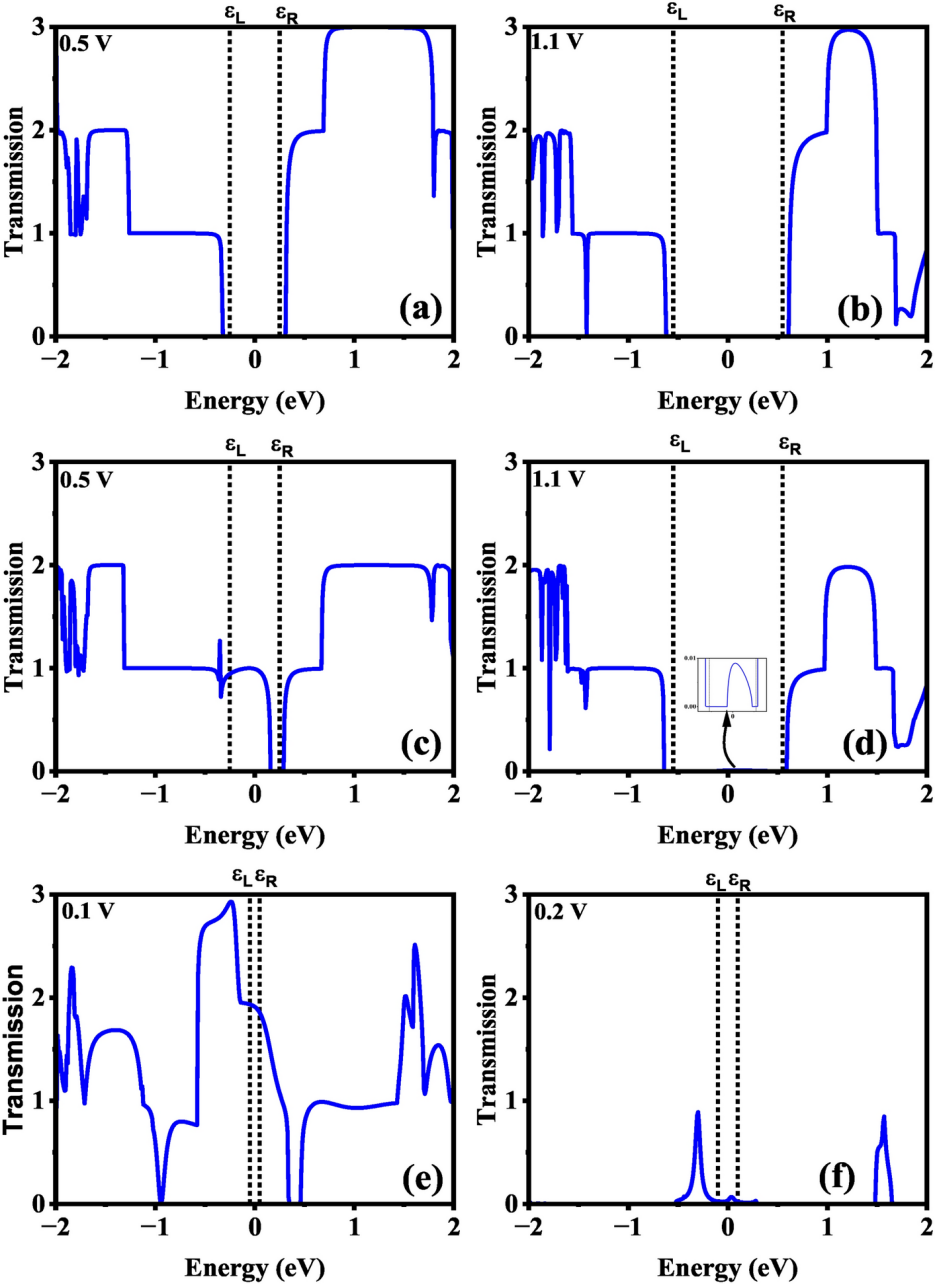
Transmission spectrum of the H-CGNR-H nanodevice at (**a**) $\hbox {V}_{{Peak}}$ of 0.5 V and (**b**) $\hbox {V}_{{Valley}}$ of 1.1 V, Cd-CGNR-H nanodevice at (**c**) $\hbox {V}_{{Peak}}$ of 0.5 V and (**d**) $\hbox {V}_{{Valley}}$ of 1.1 V, and Cd-CGNR-Cd nanodevice at (**e**) $\hbox {V}_{{Peak}}$ of 0.1 V and (**f**) $\hbox {V}_{{Valley}}$ of 0.2 V for $\hbox {N}_Z$=8 respectively.

To delve deeper into the NDR mechanism, Fig. 6 illustrates the bias-dependent transmission spectrum of H-CGNR-H, Cd-CGNR-H, and Cd-CGNR-Cd nanodevices respectively. The likelihood of carrier transmission across the scattering channel “S” region is represented by the transmission spectrum. The transmission spectrum’s area within the energy bias window ($\pm \hbox {eV}_b$) at a given applied $\hbox {V}_b$ directly relates to carrier transmission between electrodes. Moreover, controlling electron localization or delocalization within energy windows can be utilized to regulate the NDR effect.

**Fig. 7:**
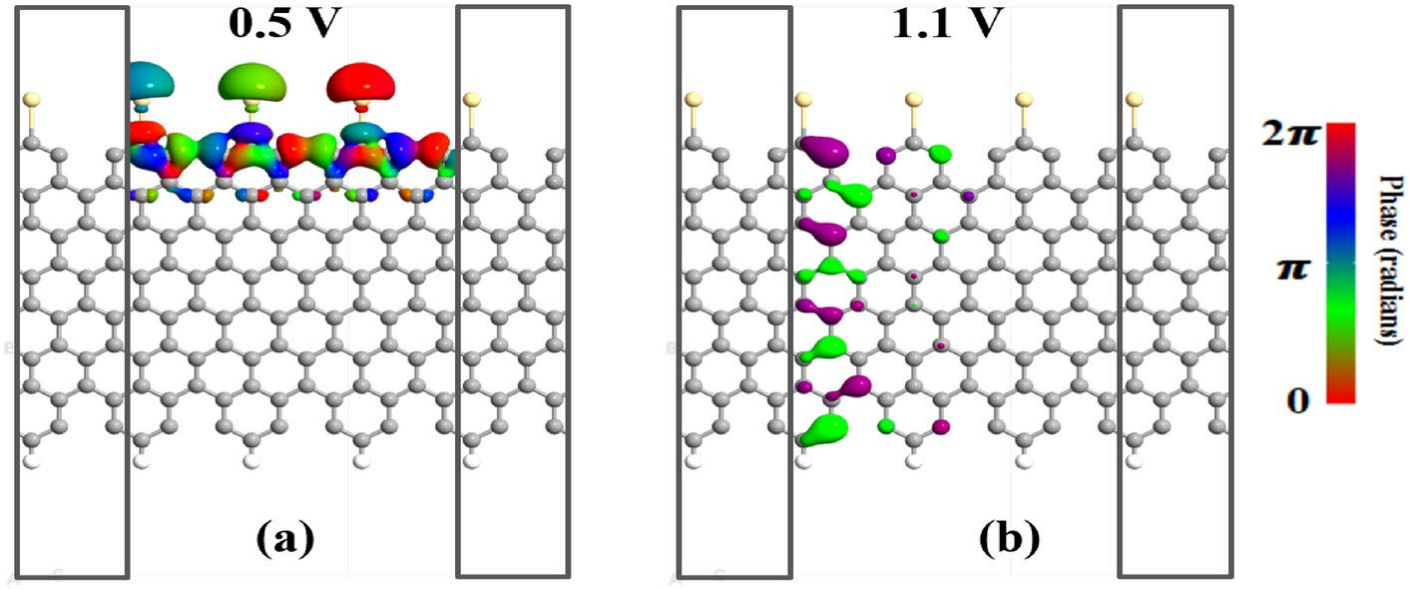
Transmission eigenchannel of the Cd-CGNR-H nanodevice for $\hbox {N}_Z$ = 8 under: (**a**) $\hbox {V}_{{Peak}}$ of 0.5 V and (**b**) $\hbox {V}_{{Valley}}$ of 1.1 V.

The transmission spectra of the H-CGNR-H device are shown in Fig. 6a,b, illustrating the absence of transmission carriers within the bias window. This results in negligible current, as depicted in Fig. 5. In contrast, significant transmission regions within the energy bias window are observed for the Cd-CGNR-H and Cd-CGNR-Cd devices, as shown in Fig. 6c–f. For the Cd-CGNR-H device, when the increases beyond 0.5 V, the transmission area within the bias window diminishes, leading to reduced current due to a limited number of carriers passing through, which correlates with the NDR effect observed in the I–V curve shown in Fig. 5 in manuscript. Similarly, for the Cd-CGNR-Cd device, larger transmission is observed at $\hbox {V}_b$=0.1 V whereas smaller transmission is evident at $\hbox {V}_b$=0.2 V. Furthermore, Fig. 7 showcases the transmission-eigenstates of the Cd-CGNR-H device. The dispersion of the device DOS along the Cd-passivated cove edge of the CGNR at 0.5 V is revealed upon examination of Fig. 7. This indicates the facilitation of carrier transmission from the “L” electrode to the “R” electrode by Cd-passivated cove edge atoms. The iso-surface value of 0.20 ${\text{\AA }}^{-3/2}eV^{-1/2}$ is upheld for the transmission-eigenstates plot at 0.5 V. Furthermore, at a bias of 1.1 V, a noticeable reduction in carrier dispersion is observed, in line with the transmission spectrum results depicted in Fig. 6. This decrease leads to a lower current magnitude and, consequently, results in the occurrence of the NDR phenomenon. The iso-surface value of 0.20 ${\text{\AA }}^{-3/2}eV^{-1/2}$ is also maintained for the transmission-eigenstates plots at 1.1 V.

**Fig. 8:**
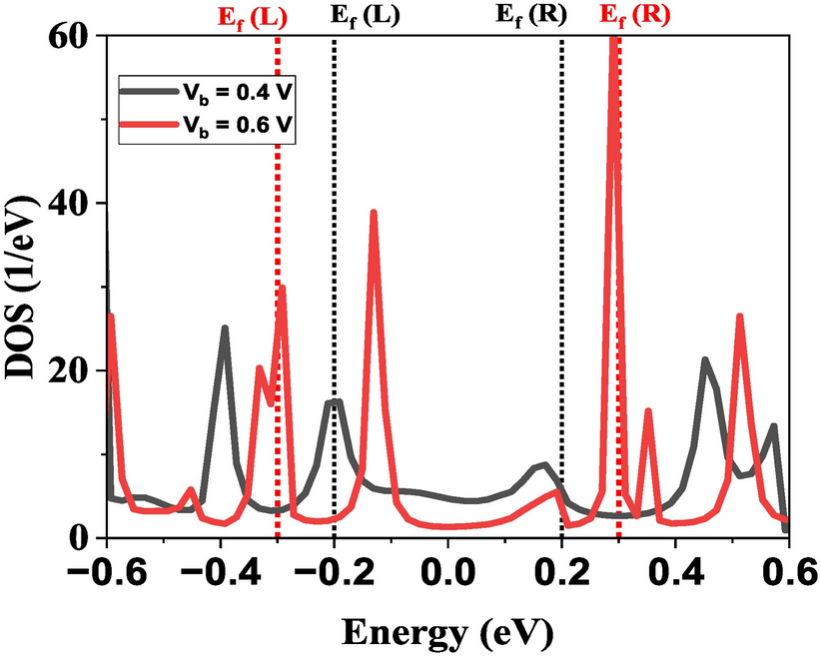
The calculated device DOS for the Cd-CGNR-Cd nanodevice for $\hbox {N}_Z$ = 8 at 0.4 V and 0.6 V.

Moreover, the multiple PVCR peaks observed in Cd-CGNR-Cd stem from a combination of localized states, scattering effects, and transmission modulation. The introduction of Cd atoms at the CGNR edges is likely to create localized states near the Fermi level, causing resonant tunneling at specific biases. To provide the detailed insight into the mechanism behind the multiple NDR peaks, we conducted bias device density of states (DDOS) calculations at key bias voltages corresponding to peak and valley currents, specifically at $\hbox {V}_b$ = 0.4 V (peak) and $\hbox {V}_b$ = 0.6 V (valley) as shown in Fig. 8. Our results reveal significant variation in the DDOS at these bias windows, shedding light on the current modulation observed in the I–V characteristics. At $\hbox {V}_b$ = 0.4 V, which corresponds to a peak in the I-V curve, we observe an elevated density of states within the bias window, − 0.2 eV to + 0.2 eV. This higher DDOS in the bias window enhances electron transmission across the device, contributing to the observed increase in current. However, at $\hbox {V}_b$ = 0.6 V, which corresponds to a valley, the DDOS within the bias window (− 0.3 eV to + 0.3 eV) is considerably reduced. This reduction limits the carrier transmission, resulting in a lower current. This modulation of DDOS with applied bias supports the emergence of multiple NDR peaks, as the changing DDOS within the bias window influences the transport properties of the Cd-passivated CGNR. The alternating DDOS values at different biases create the fluctuating current responses seen as multiple NDR peaks. These findings are consistent with similar studies^45^, where DDOS variations, particularly around edge states, have been correlated with NDR behavior. These localized states modified by the applied bias, resulting in multiple PVCR peaks as the system periodically aligns with these resonances. Furthermore, Cd atoms act as scattering centers, contributing to variations in electron flow across the CGNR. As the bias increases, these scattering sites intermittently disrupt transport, further enhancing the stepwise current drops associated with the NDR effect. The semiconductor-to-metal transitions and changes in the band alignment in the Cd-passivated structures, evidenced by the band structure and PDOS analysis as shown Figs. 3 and 4, reveal that edge atoms facilitate or hinder carrier transmission, depending on the bias. Together, these findings provide a deeper understanding of the role of Cd in tuning the transport characteristics of CGNRs and suggest potential applications in nanoelectronics, where controllable NDR characteristics are desirable.

**Table 4:** A comparison of the PVCR of the proposed ultra-scaled nanodevice based on Cd-CGNR with the state-of-the-art.

S.No.	Devices	Bond length (Å)	Electronic property	Peak voltage ($\hbox {V}_{{Peak}}$)	Valley voltage ($\hbox {V}_{{Valley}}$)	PVCR
1	Planar strained graphene nanoribbon (GNR)^46^	1.42	Semiconductor	0.09 V	0.14 V	5.63
2	Planar zigzag GNR (ZGNR) junction^47^	1.42	NR	1.50 V	2.50 V	10.00
3	Planar armchair edge oriented GNR^48^	1.42	NR	0.09 V	0.22 V	21.00
4	Planar armchair edge oriented GNR with antidot^49^	1.42	Semiconductor	0.06 V	0.09 V	29.40
5	Planar BN-graphene heterostructure^45^	1.42	Semiconductor	0.50 V	1.00 V	13.00
6	Puckered phosphorene-doped AGNR^50^	1.42	Metal	0.60 V	1.20 V	5.40
7	Puckered black phosphorus^51^	2.22	Semiconductor	0.70 V	0.90 V	9.00
8	Sandwich $\hbox {MoS}_2$ nanoribbon^52^	2.42	Semiconductor	0.23 V	0.71 V	16.23
9	Buckled silicene nanoribbon^53^	2.28	Semiconductor	0.30 V	0.70 V	3.09
10	Planar zigzag ZrSe2 nanoribbon^54^	2.72	Semiconductor	0.81 V	0.91 V	1.50
11	Planar armchair ZrSe2 nanoribbon^54^	2.66	Semiconductor	0.77 V	0.90 V	19
12	**Planar Cd-CGNR-H (Present work)**	1.42	Metal	0.50 V	1.1 V	**53**.**7**

*NR - Not reported

The results from the Cd-CGNR-H device’s NDR are extensively compared to the latest NDR research see Table 4. Furthermore, edge passivation would be an easier approach compared to doping. The comparison in Table 4 shows that CGNRs display larger PVCR within a narrow $\hbox {V}_b$ range.

## Conclusion

In conclusion, our comprehensive analysis employing band structure and density of states calculations, has unraveled the complex interplay among the edge passivation, Cd atoms, and the electronic behavior in cove-edge graphene nanoribbons. Observing bond length reconstructions at the cove-edge atoms in the studied CGNRs confirms their thermal stability, as evidenced by the obtained binding energy. The exploration of semiconductor-to-metal transitions, localized energy levels, and alterations in DOS profiles signifies the immense potential for customizing the electronic properties using strategic passivation methodologies. Highlighting the semiconducting nature of H-passivated CGNRs, with a bandgap of 0.14 eV, stands in contrast to the metallic behavior observed in Cd-passivated CGNRs due to free dangling bonds across bare cove-edge atoms. Notably, the revelation of NDR features in the cove-edge devices through their transport characteristics, with Cd-CGNR-H showcasing NDR across a broad bias range and Cd-CGNR-Cd within a relatively narrow bias range 0.1 V–0.2 V, underscores the influence of Cd-passivated atoms on the carrier transmission owing to the higher electronegativity of Cd atoms. The notably high PVCR value of 53.7 for the Cd-CGNR-H device holds promise for the design and development of advanced nanoelectronic devices, harnessing the unique electronic functionalities of passivated cove GNRs, thereby paving the way for innovative avenues in nanoelectronics device design. As future work, multi-element passivation techniques could be explored to enhance the tunability of CGNRs’ electronic properties. Additionally, device modeling along with experimental efforts in incorporation of passivants of the CGNRs edges will be critical for understanding their impact on device performance. Overall, this study lays the groundwork for innovative applications in real-world devices, including transistors and memory components, advancing the field of nanoelectronics.

## Data Availability

The datasets used and/or analysed during the current study available from the corresponding author on reasonable request.
